# Broiler farming practices using new or re-used bedding, inclusive of free-range, have no impact on *Campylobacter* levels, species diversity, *Campylobacter* community profiles and *Campylobacter* bacteriophages

**DOI:** 10.3934/microbiol.2024002

**Published:** 2024-01-16

**Authors:** Helene Nalini Chinivasagam, Wiyada Estella, Damien Finn, David G. Mayer, Hugh Rodrigues, Ibrahim Diallo

**Affiliations:** 1 Department of Agriculture and Fisheries, Eco Sciences Precinct, Dutton Park QLD 4102, Australia; 2 Department of Agriculture and Fisheries, Biosecurity Sciences Laboratory, Coopers Plains QLD 4108

**Keywords:** *Campylobacter*, bacteriophages, broiler, litter, free-range, re-use

## Abstract

A multi-stage option to address food-safety can be produced by a clearer understanding of *Campylobacter*'s persistence through the broiler production chain, its environmental niche and its interaction with bacteriophages. This study addressed *Campylobacter* levels, species, genotype, bacteriophage composition/ levels in caeca, litter, soil and carcasses across commercial broiler farming practices to inform on-farm management, including interventions.

Broilers were sequentially collected as per company slaughter schedules over two-years from 17 farms, which represented four commercially adopted farming practices, prior to the final bird removal (days 39–53). The practices were conventional full clean-out, conventional litter re-use, free-range–full cleanout and free-range–litter re-use. Caeca, litter and soil collected on-farm, and representative carcases collected at the processing plant, were tested for *Campylobacter* levels, species dominance and *Campylobacter* bacteriophages. General community profiling via denaturing gradient gel electrophoresis of the *flaA* gene was used to establish the population relationships between various farming practices on representative *Campylobacter* isolates. The farming practice choices did not influence the high caeca *Campylobacter* levels (log 7.5 to log 8.5 CFU/g), the carcass levels (log 2.5 to log 3.2 CFU/carcass), the *C. jejuni*/*C. coli* dominance and the on-farm bacteriophage presence/levels. A principal coordinate analysis of the *flaA* distribution for farm and litter practices showed strong separation but no obvious farming practice related grouping of *Campylobacter*. Bacteriophages originated from select farms, were not practice-dependent, and were detected in the environment (litter) only if present in the birds (caeca).

This multifaceted study showed no influence of farming practices on on-farm *Campylobacter* dynamics. The significance of this study means that a unified on-farm risk-management could be adopted irrespective of commercial practice choices to collectively address caeca *Campylobacter* levels, as well as the potential to include *Campylobacter* bacteriophage biocontrol. The impact of this study means that there are no constraints in re-using bedding or adopting free-range farming, thus contributing to environmentally sustainable (re-use) and emerging (free-range) broiler farming choices.

## 1. Introduction

Poultry farming is efficient, thereby providing nutritional security to many nations, with global production reaching 133.4 million tonnes in 2020. However, it leaves a significant environmental footprint, requiring sustainable waste management practices, including litter management [1]. In Australia, conventional broiler farming is the dominant practice. In a climate of dwindling bedding resources, litter re-use, supported by pathogen reduction between cycles, contributes to sustainable farming [2]. Whilst litter re-use is adopted across four Australian states [3], in Queensland it has been a long-term practice for over 30 years [4]. Litter is also re-used in other countries such as the USA [5],[6]. Previous studies have investigated the Australian practice of partial litter re-use, including a detailed analysis of in-shed management with a focus on both *Campylobacter* and *Salmonella* [4],[7]–[9]. In Australia, free-range chicken meat and egg production is becoming more popular due to the demand for pasture/range raised birds [10], and in other countries because of emerging consumer preferences [11]. An understanding of microbial pathogens within free-range systems can enhance environmental sustainability of pastured poultry farming [12].

To optimise food-safety, assessments on pastured poultry farming practices with a focus on soil, faeces and carcasses have contributed to addressing *Campylobacter* prevalence in US poultry [13]. Additionally, identifying the gaps in knowledge in pathogen distribution associated with the complex supply chains of pasture raised flock in comparison to conventional systems is vital to support risk-based decisions and management [14]. In Australia, a mix of practices such as conventional (new bedding), re-use, and free-range (with new or re-used bedding) can occur within a single integrator. Given the different natures of their operations, alongside a potential to locally spread pathogens, both sustainable and alternative broiler farming options can attract food-safety concerns.

*Campylobacter*, a major human pathogen, colonises the chicken gut [15] and is a primary causative agent of human illness [16]. In the European Union, broiler meat was the key single source responsible for human campylobacteriosis (47% in 2015) [17]; in New Zealand, poultry sources were linked to 75–90% of that country's campylobacteriosis cases [18]. *Campylobacter jejuni* is responsible for most human gastroenteritis cases (~95%) [19]; although *C. coli* contributes to a minority of human illness, the health burden can be significant [20]. Moreover, other food commodities along with poultry meat were identified as main routes of infection; thus , there is a need to address *Campylobacter* reduction across the general agroecosystem [21].

In Australia, campylobacteriosis is the highest contributor to gastro-intestinal diseases [22]. In the USA, estimates suggest that over 1.3 million people are affected by campylobacteriosis annually [23]. Whilst the true incidence of *Campylobacter* associated human illness is underestimated, the notification rates can range from 29 cases (Canada) to 135.3 cases (New Zealand) per 100,000 population [24]. Irrespective of the international focus on reducing campylobacteriosis, the hygienic measures that have been successful in reducing *Salmonella* in poultry have not been so with *Campylobacter* due to its differing biology [25].

*Campylobacter jejuni* and *C. coli* are well adapted to the ecological niche of the avian gut; for effective control strategies, these organisms need to be better understood within poultry systems [26]. The vulnerability of the chicken gut to pathogen colonisation is linked to the changing gut microbiota influenced by microorganisms originating from artificial farming environments, which are a feature of modern poultry farming [27]. Microbial succession in the chicken gut plays a contributory role towards *Campylobacter* emergence [28]. As a primary reservoir for *Campylobacter*, poultry shows no signs of disease [29] or inflammatory responses and *Campylobacter* has a commensal interaction with the bird [30]. Chickens commonly remain *Campylobacter*-free until around 2–3 weeks of age (or mid-cycle); then, they are rapidly colonized with doses as low as 10 CFU (to a maximum of 10^9^ CFU/g) and continue to remain colonized throughout their lifespan [26].

In contrast to the above, the birds remained *Campylobacter*-free during an entire cycle of ~55 days when tested simultaneously across three different litter practices (including re-use) [9]. Recent studies have shown *Campylobacter* DNA in the feces of commercially reared meat chicks less than eight days of age suggesting colonisation of a much younger bird [31]. *Campylobacter* can be present in the breeder flock and eggs prior to environmental transmission [32]; nevertheless, vertical transmission in broilers remains controversial [33]. *Campylobacter* can originate from on-farm sources such as wildlife, birds and water in broiler environments [34]. Thus, there is a need for an overall understanding of the on-farm *Campylobacter* ecology in commercial broiler flocks [35].

Several studies have addressed epidemiological links to control the colonisation of commercial broilers with a focus on on-farm biosecurity [36]–[38], including in free-range operations [11]. However, the outcomes of on-farm biosecurity measures to control *Campylobacter* have been inconsistent to date [31]. As of 2020, the European Food Safety Authority [39] highlighted the effectiveness of biosecurity alongside any associated complications, which depends on individual control options driven by interrelated local factors. Moreover, other factors such as local geography and hours of sunshine and rain, both driven by seasonality, can impact *Campylobacter* colonisation in broilers [40]. On its own, biosecurity does not decrease *Campylobacter* colonisation in broilers; however, with the addition of basic on-farm hygiene forms a basis for interventions that target a reduction in *Campylobacter* levels, to be effective [41]. Interventions that target *Campylobacter* reduction in the caeca should be a priority for on-farm control policy [42].

In comparison to conventionally raised birds, the campylobacters originating from environmentally exposed free-range birds can also be challenged by bacteriophages, which are attractive biocontrol agents sourced from farmed environments [43]. The use of natural alternatives such as bacteriophages as interventions are gaining ground and are driven by consumer perceptions [44]. They are already widespread in food and water and thus are regularly consumed; moreover, their host specificity makes them safe biocontrol agents [45]. The ultimate success of bacteriophage biocontrol is consumer acceptance; the use of bacteriophages that already exist in chickens is likely to gain acceptance rather than from other sources [46].

Bacteriophages play an important role in shaping microbial ecosystems [47] and constantly evolve to overcome host barriers to infection [48]. Both *Campylobacter* and *Campylobacter* bacteriophages naturally occur together in a predator – prey relationship and are a natural pathogen reduction food-safety strategy [49]. The use of bacteriophages against *Campylobacter* in poultry has been previously reviewed [50],[51]. In vitro studies have demonstrated a reduction of *Campylobacter* via poultry sourced bacteriophages [52],[53]. On-farm studies suggest that a maximum reduction of *Campylobacter* may be possible at the plant by introducing bacteriophages 1–4 days prior to slaughter [54]. A recent, proof-of-concept Australian study using a bacteriophage cocktail to reduce *Campylobacter* levels in the chicken gut demonstrated a 2-log reduction in the caeca of commercially farmed broilers on farm A; however, indigenous bacteriophages also contributed to *Campylobacter* reduction on farm B in both control and test birds, either before or concurrently, with the bacteriophage intervention [55].

The bacteriophages that originated from the current study formed the original 19-candidate phage panel from which the cocktails on farm A and B were derived [56]. This work was followed by a detailed analysis of select bacteriophages with a focus on biocontrol [57]. Introducing *Campylobacter* bacteriophages had no impact on the microbiota structure of the chicken gut other than reducing the targeted *C. jejuni*, thus making it a safe biocontrol option [58]. Consequently, there is a need for a comprehensive understanding of the on-farm *Campylobacter*–bacteriophage relationships to aid in bacteriophage biocontrol, which is also a focus of the current study.

*Campylobacter* survives well from poultry farms, to slaughter and to the final product, thereby suggesting it has either adaptive responses (that remain elusive) or protected environmental niches throughout the poultry process chain or both [59]. Since it is not possible to eliminate *Campylobacter* across the food chain, targeting on-farm intervention strategies to reduce the quantitative burden in flock on-farm is the most effective point of control [60]. A review of the European Union control strategy for *Campylobacter* in the broiler meat chain suggests the need for an effective comprehensive risk management strategy that addresses the whole process chain, which is supported by evidence-based risk assessment studies to achieve both economic and public health impacts [24]. Currently, no single control method is known to fully control *Campylobacter* contamination comprehensively in the broiler industry [59], in which *Campylobacter* continues to remain a cause of concern [61].

The current study builds upon already undertaken research to formulate an informed basis for on-farm *Campylobacter* risk management. To be of practical relevance and facilitate industry uptake, the study focused on four Australian commercial broiler farming practices – conventional and free-range – with either full or partial litter removal. A quantitative approach was adopted to assess the bird (caeca), the farming environment (i.e., litter, soil) and the processing plant (carcasses) simultaneously over two years across 17 commercial farms and plants. More specifically, the *Campylobacter* levels, the species diversity, the *Campylobacter* populations (based on genetic similarity in the *flaA* short variable region (SVR) phylogenetic marker) and the *Campylobacter* bacteriophages were studied. The aim of the study is to contribute to on-farm *Campylobacter* risk management alongside the potential for bacteriophage biocontrol. The overall outcome will ultimately support both safe and sustainable broiler farming.

## 2. Materials and methods

### 2.1. Animal ethics

Animal Ethics approval for the entire study was granted by the Department of Agriculture and Fisheries Animal Ethics Committee (AEC Proposal Reference Number SA 2011/11/372).

### 2.2. Farms and litter practices

**Figure 1.**
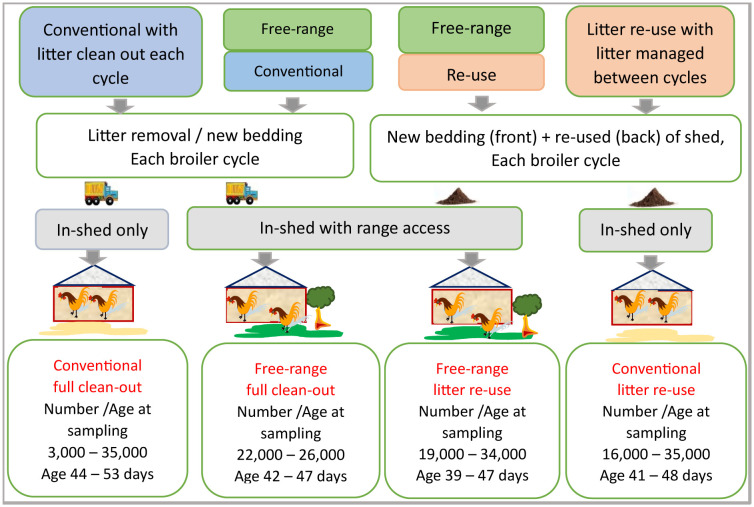
Schematic of experimental design.

To be of practical relevance and facilitate industry uptake, the study was performed in consultation with the industry. Seventeen farms across three regions of outer Brisbane, Queensland, Australia representing four farming practices (Figure 1) from two major companies were selected for the study over two years. The selection of farms was based on their sequential availability for slaughter as per the routine company schedule, and thus is a natural representation of dominant practices. These farms were sampled at 1–2 months intervals (a complete farming cycle can last up to 55 days). Eleven farms were sampled in year 1 and 13 farms in year 2. Based on the 2-year sampling plan, seven farms from the original set of farms were sampled in year 2, thus leading to the accommodation of a total of 24 farm samplings. Sampling occurred just prior to the final bird removal (a company decision based on market needs) when the birds were aged 39–53 days. The numbers of birds remaining on-farm varied based on thinning that occurred prior to the final removal on all farms and ranged from 16,000–35,000 on 21 of the farms and 3000–5000 for rest of the farms at the time of sample collection. The farm types were conventional full clean-out (CN_FC), conventional litter re-use (CN_RU), free-range–full cleanout (FR_FC) and free-range–litter re-use (FR_RU). The age and number of birds that remained at sampling for each practice are presented in Figure 1. The Australian litter re-use practice, which is a partial litter re-use, has been previously described [9] and includes a litter pile-up of around 4–5 days between cycles. Free-range (i.e., bird access to the range, when fully feathered) was adopted as [62].

### 2.3. Farm Sampling

For sample collection from chickens and litter, the shed was categorized into four equal segments (i.e., C1, C2, C3 and C4) based on the number of bays (shed struts). The sections were used as a guide to randomly collect chickens and litter covering the area across each shed segment. Thirty-two chickens and 32 litter samples were randomly collected per shed (i.e., eight chickens or litter samples per shed segment). The representative birds were euthanized in a humane manner (AEC Proposal Reference Number SA 2011/11/372) on farm and the caeca were aseptically removed. The bird caeca samples (eight caeca) from each segment of the shed were mixed well to form a single uniform composited sample (e.g., C1, for caeca from segment 1). Thus, four composite caeca samples (segments C1–C4) were prepared per shed at each sampling.

### 2.4. Litter and soil collection

Litter was collected to a depth of 40 cm over an area of 400 cm^2^, as described in [8]. The litter from shed segment 1 with 2 and 3 with 4 were composited to form two composite litter samples, L1 and L2. The soil samples were collected either on a single side of the shed (non-free range) or range (free-range) in a random manner. An aseptic stainless steel soil sampler was inserted to a depth of 4–5 cm into the soil and a total of eight soil core samples were collected per segment (half of the area, S1 or S2). All caeca, litter and soil samples were transported chilled and stored at 4 °C on arrival at the laboratory. *Campylobacter* was tested within 24 h.

### 2.5. Campylobacter levels in caeca, litter and soil

Twenty-five grams of caeca, litter or soil was weighed into 225 mL of Preston broth without antibiotics (Nutrient broth No2 with 5% lysed horse blood). A stomacher (Smasher AESAPI064) was used to macerate the caeca for one minute and a stick blender (Barmix) was used to blend the litter for one minute. All soil samples were shaken for 15 minutes and allowed to settle. *Campylobacter* levels in the caeca, litter and soil were determined via direct plating, where serial dilutions for each were directly plated onto *Campylobacter* blood-free selective agar CCDA (Oxoid) with selective supplement (Oxoid, SR0155) and incubated at 37 °C for 48 h under micro-aerobic conditions using Campygen (Oxoid, CN0025A). *Campylobacter* levels following enumeration are presented as log CFU/g.

### 2.6. Campylobacter levels in carcasses

The carcasses representing each farm were generally available from the processing plant either one- or two-days post farm sampling, depending on the final bird removal (and were alive until reaching the plant). Immediately upon slaughter, the carcasses were removed, and rinses were carried out at each company laboratory as per the company protocol. Briefly, the carcasses were either mechanically or manually rinsed in 200 mL of diluent (0.1% peptone water) (based on company practice), transported chilled to the laboratory and tested within 24 hours. A total of 15 carcasses were randomly removed from the processing plant pertaining to the relevant batch of chickens and each carcass was individually tested, resulting in 15 rinses (R) per farm, which were averaged and presented as log CFU/carcass.

### 2.7. Campylobacter isolates for species and flaA-SVR grouping

Across each of the 24 farm samplings over the two years, a total of ~50 randomly selected *Campylobacter* isolates per farm were picked from the three CCDA plates, from which the bacterial count was derived for that sample. Thus, the colonies were taken from the lowest countable dilution (which has the higher number of well separated colonies with dominant and lesser dominant colonies) and two higher dilutions (separated colonies and dominant colonies), thus representing colony diversity. More specifically, a total of 20 isolates were taken from the caeca (i.e., C1, C2, C3 and C4) and 10 isolates from the carcass rinses (R1, R2). Similarly, 10 isolates each were picked from the litter (L1, L2) and the soil (S1, S2) A total of 812 isolates were streaked for purity and stored for further analysis.

### 2.8. Campylobacter species identification by PCR

All isolates were confirmed as either *C. jejuni* or *C. coli* using the optimized rapid duplex real-time PCR [63]; for select *C. coli* isolates that could not be differentiated using the real-time PCR of [63], the real-time PCR of [64] was used. All these *C. coli* isolates came from a single farm. Over the two-year period, the species identity of a total of 812 isolates were performed using real time PCR.

### 2.9. Denaturing gradient gel electrophoresis (DGGE)

Denaturing gradient gel electrophoresis (DGGE) was used as a molecular typing technique for the *flaA*-SVR, as previously described [65],[66]. DGGE was performed with a universal mutation detection system (Bio-Rad Laboratories, Sydney, Australia) and polyacrylamide gels were silver stained for visualization, as described by [67] and [68]. Stained gels were scanned with an Epson Perfection U700 Photo scanner and imported to Adobe Photoshop Elements v 3.0 (Adobe Systems Inc., San Jose, CA, USA). Images were analysed with Bionumerics v 6.0.1 (Applied Maths, Sint-Marten-Latems, Belgium). Band profiles were compared with weighted Pearson correlation and the profiles were considered as belonging to the same *flaA*-SVR group with a similarity index greater than 0.85. A principal coordinates analysis and an analysis of similarity (ANOSIM) were performed in R v 3.5.2 [69] with the vegan package [70]. A total of 757 representative *Campylobacter* isolates were analysed.

### 2.10. Enumeration of Campylobacter bacteriophages

The bacteriophage levels were analyzed similarly to *Campylobacter* levels, as previously described. More specifically, the composite samples representing segments C1, C2, C3, C4 for the caeca, L1 and L2 for the litter and S1 and S2 for the soil were tested across all 24 farm samplings. Enumeration via direct plating for the caeca was based on the methodology in [71] with slight modifications. Ten grams of caeca was weighed into 90 mL of Salt Magnesium (SM) buffer (100 mM NaCl; 8 mM MgSO4.7H2O; 0.01% gelatin; 50 mM Tris-HCl, pH7.5) then stomached (Smasher AESAPI064) for one minute, followed by gentle shaking at 4 °C overnight on a platform shaker. The samples were distributed into micro-centrifuge tubes, centrifuged at 15,000 x *g* for 5 min, chilled for 5 min, then centrifuged again at 15,000 x *g* for 5 min. The supernatant was decanted to a new tube and filtered using membrane filtration with a 0.22 µm pore size filter (low DNA binding, Minisart; Sartorius) and stored for bacteriophage enumeration.

The samples for bacteriophage enumeration were prepared following enrichment using *C. jejuni*, NC3142 (a farm isolate from litter) and *C. coli*, NC2934 (a farm isolate from caeca) grown in Nutrient broth No. 2 (NB2) with 5% lysed horse blood (LHB) (v/v) with overnight incubation at 42 °C, as described in [55]. For bacteriophage enumeration (direct or enriched), a mixture of 100 µL sample plus 200 µL of 10^8^ CFU/mL *C. jejuni* PT 14 (NCTC 12662) host was aerobically incubated at 42 °C for 30 min. Then, this mixture was added to 5 mL of a 0.6% agar overlay, which was poured on top of a New Zealand casamino yeast medium (NZCYM) base plate (NZCYM broth plus 1% bacteriological agar) and allowed to settle for around 30 minutes. The plates were incubated at 42 °C for 24 hours under micro-anaerobic condition, as described in [55]. Bacteriophage levels were enumerated from countable plates, and the levels are presented as log PFU/g. The genome size of select bacteriophages was determined using pulsed field gel electrophoresis (PFGE), as described by [71].

### 2.11. Statistical analysis

*Campylobacter* levels in the caeca, litter, soil and carcass rinses were separately analysed in [72] using an unbalanced analysis of variance (ANOVA) model. The 5% probability level (*P* < 0.05) was adopted for statistical significance. Levels were positively skewed with heterogeneous variances; therefore, they were log_10_-transformed prior to the analysis. The fixed effects were the farming practices (conventional full clean-out, conventional re-use, free-range full clean-out and free-range re-use; both as a four-level factor and as a two-by-two factorial), the three regions, and the years (year 1 and 2). The farms were taken as the random effect, with the multiple locations within each farm as subsamples. Interactions were screened but were not included in the final model, as none were significant.

The species diversity between *C. coli* and *C. jejuni* in the caeca, carcass, litter and soil were investigated. The proportions of *C. jejuni* in these samples were analysed in [72] using a generalized linear model [73] under the Binomial distribution and logit link. The caeca and carcass data showed a high variation; therefore, for these, an over-dispersed model was adopted. The fixed and random effects were the same as listed above. Similarly, interactions were screened but dropped from the final model, as none were significant.

## 3. Results

### 3.1. Campylobacter levels in caeca, litter, soil and carcasses

**Figure 2.**
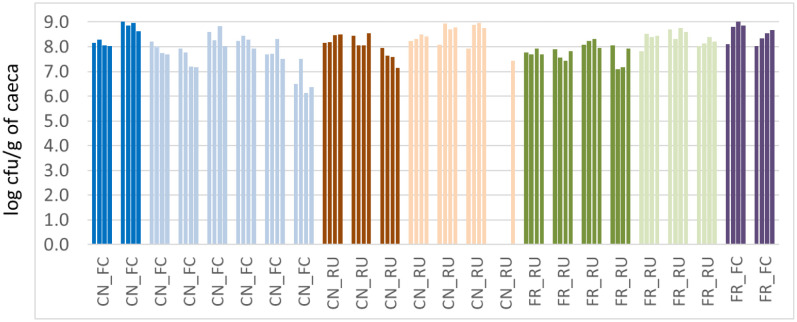
*Campylobacter* levels in caeca across shed sections (C1, C2, C3, and C4) from 24 farm samplings in Year 1 (darker shade) and Year 2 (lighter shade) for conventional full clean-out (CN_FC), conventional re-use (CN_RU), free-range full clean-out (FR_FC) and free-range re-use (FR_RU).

Irrespective of litter practice, the *Campylobacter* levels (C1, C2, C3, C4) in the caeca ranged from around a minimum of log 6.1 to a maximum of log 9.0 CFU/g across both years, as shown in Figure 2. Most importantly, the *Campylobacter* levels were generally uniform across a whole shed (as represented by shed sections C1, C2, C3, C4) across all 24 farms tested. However, one CN_RU farm (DK_13) proved to be an exception, with *Campylobacter* only being detected in one quarter of the shed but with bacteriophages present across the whole shed (described under bacteriophage section). Irrespective of being only detected in one end (i.e., segment, C4), the levels (log 7.2 CFU/g) were consistent with other farms.

**Figure 3.**
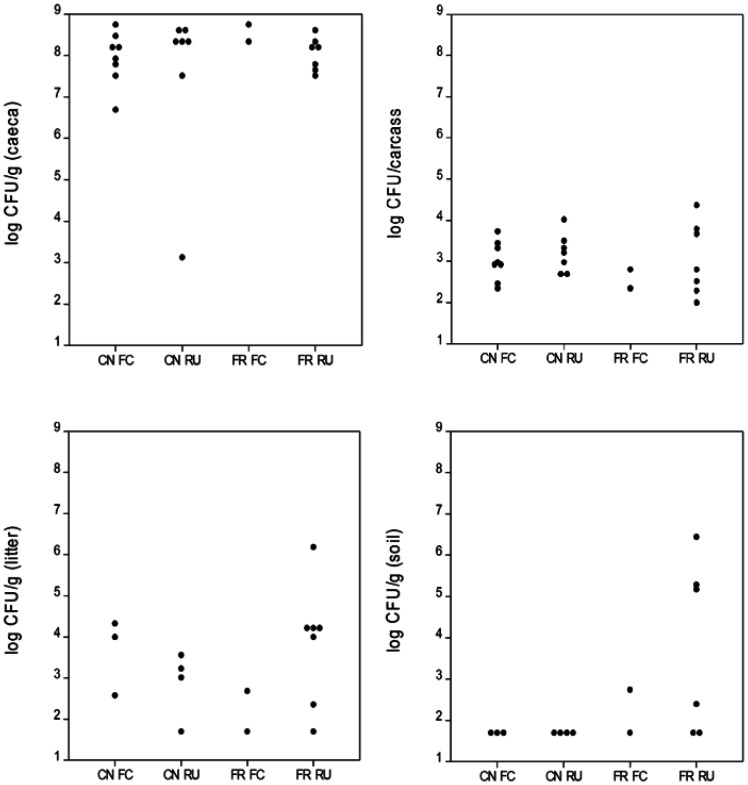
*Campylobacter* levels in caeca (log CFU/g), carcass (log CFU/carcass), litter (log CFU/g) and soil (log CFU/g) for conventional full clean-out (CN_FC), conventional re-use (CN_RU), free-range full clean-out (FR_FC) and free-range re-use (FR_RU).

Figure 3 presents the comparative *Campylobacter* levels across the four litter practices (i.e., conventional full clean-out, CN_FC, conventional re-use, CN_RU, free-range full clean-out, FR_FC and free-range re-use, FR_RU) for the caeca, litter, soil and carcasses across both years. For the caeca and carcasses, the *Campylobacter* levels across litter practices showed a decreased variability, ranging from ~log 7.0–to log 9.0 CFU/g (caeca) and log 2.0–to log 4.0 CFU/carcass (carcasses).

Table 1 presents probability levels (*P*) and the mean *Campylobacter* levels for the caeca, litter, soil and carcasses for the four litter practices. There were no significant differences in the *Campylobacter* levels in the caeca, carcasses, litter and soil across the litter practices. Additionally, there were no significant differences between the regions or years for the caeca, litter, soil and carcasses (means not presented).

**Table 1.** Mean *Campylobacter* levels for caeca, litter, soil (log CFU/g), carcass (log CFU/carcass) for conventional full clean-out (CN_FC), conventional re-use (CN_RU), free-range full clean-out (FR_FC) and free-range re-use (FR_RU), probability levels (*P*) of the litter practice differences, and average standard errors (s.e.).

	Caeca, log CFU/g	Litter, log CFU/g	Soil, log CFU/g	Carcass, log CFU/carcass
*P*	0.74	0.33	0.13	0.66

CN_FC	7.938	3.627	1.700	3.020
CN_RU	7.562	2.858	1.700	3.209
FR_FC	8.546	2.220	2.200	2.572
FR_RU	8.033	3.843	3.791	3.071

s.e.	0.534	0.652	0.794	0.284

Although there was approximately one log difference between the mean caeca *Campylobacter* levels, from log 8.5 CFU/g (free-range full clean-out) to log 7.6 CFU/g (conventional re-use), these levels were not statistically significant, as shown in Table 1. Irrespective of the high *Campylobacter* levels in the caeca, the mean carcass levels at the end of the processing line across years 1 and 2 ranged from log 2.6 CFU/carcass (free-range full-clean-out) to log 3.2 CFU/carcass (conventional re-use), in which levels across all four litter practices were not significantly different. There were some instances of high *Campylobacter* levels in the soils of free-range with litter reuse farms (log 5.0–7.0 CFU/g, Figure 3); however, the overall *Campylobacter* levels for soil and litter across farms were not statistically significant.

### 3.2. Campylobacter species diversity

**Figure 4.**
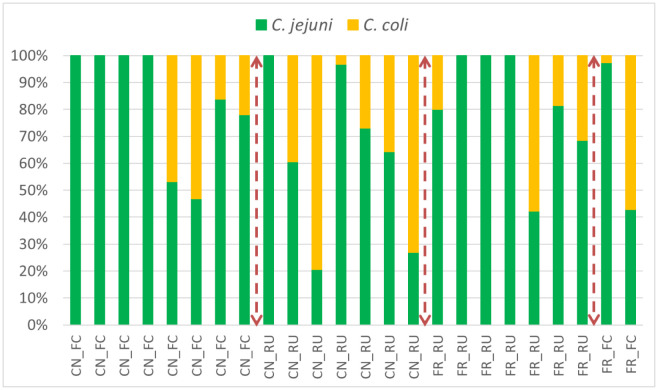
Percentage of *C. jejuni* and *C. coli* (Year 1–Year 2) from 17 farms (24 farm samplings, as some sampled in both years), for conventional full clean-out (CN_FC), conventional re-use (CN_RU), free-range full clean-out (FR_FC) and free-range re-use (FR_RU) (total isolates 812).

Figure 4 illustrates the relative proportion (%) of *C. jejuni* and *C. coli* isolates over the two years as analysed by a real time PCR analysis. These isolates (812 in total) were collectively sourced from the caeca, litter, soil and carcasses across the individual farms and grouped according to the litter practices. Overall, *C. jejuni* represented 75% of the isolates, whilst the rest (25%) were *C. coli*. A 100% *C. jejuni* dominance was observed across multiple practices (i.e., free-range re-use, full-cleanout or conventional litter re-use) (Figure 4). In contrast, *C. coli* dominance (75–80%) was mainly observed with the litter re-use practice (Figure 4), though dominance was also distributed across other practices but to a lesser extent. In summary, *Campylobacter* species diversity (Figure 4) showed no practice driven patterns across the 24 farm samplings over the two-year period.

The effect of litter practices on *Campylobacter* species percentages was analysed for the caeca, litter and carcass. There were no significant differences in the percentage of *C. jejuni* found between the regions or years (means not presented). Table 2 shows that there was no significant effect of the litter practices on the percentage of *C. jejuni* for the caeca; however, this effect was significant for the carcass and litter. For the carcasses, conventional litter reuse (71%) and free-range re-use (71%) had significantly lower *C. jejuni* percentages than full clean-out (91%) and free-range full clean-out (95%). For the litter, conventional litter reuse (68%) had a significantly lower *C. jejuni* percentage compared to the other three farm practices (~100%).

**Table 2.** Percentage *C. jejuni* (and standard errors) for conventional full clean-out (CN_FC), conventional re-use (CN_RU), free-range full clean-out (FR_FC) and free-range re-use (FR_RU), and probability levels (*P*) of the litter practice differences.

	Caeca	Carcass	Litter
P	0.379	0.002	<0.001
CN_FC	75.3 (13.0)	91.3^a^ (3.3)	98.0^a^ (2.0)
CN_RU	48.1 (16.5)	71.1^b^ (7.0)	68.0^b^ (9.3)
FR_FC	61.5 (29.5)	94.7^a^ (3.8)	100.0^a^ (17.9)
FR_RU	84.7 (11.6)	71.4^b^ (7.1)	100.0^a^ (13.6)

*Within columns, means with different super-scripts are significantly different.

### 3.3. Campylobacter isolates

The dendrogram (Figure S1 in supplementary material) presents the analysis of *Campylobacter* isolates sourced in year 1, with clustering based on the *flaA*-SVR locus with a weighted Pearson correlation of DGGE banding profiles. As multiple gels were required to compare all 329 *Campylobacter* isolates sourced in year 1, an 85% similarity cut-off was chosen to prevent separate gels from biasing *flaA*-SVR groupings. The farm site appeared to play a greater role in separating *flaA*-SVR groups than the *Campylobacter* species or sample type. The ANOVA of *flaA*-SVR groups from caecal isolates demonstrated a highly significant difference in the numbers of isolates between *flaA*-SVR groups (*P* = 9.37 x 10^−9^). Subsequently, a total of 757 *Campylobacter* isolates across both years were analysed and represented all 24 samplings from 17 farms, and originated from the caeca, litter, soil and carcasses. These isolates were grouped into 14 different *flaA-SVR* groups, with groups 7, 10 and 14 being the most dominant (data not presented).

### 3.4. Principal Coordinate Analysis (PCoA) on Campylobacter isolates

**Figure 5.**
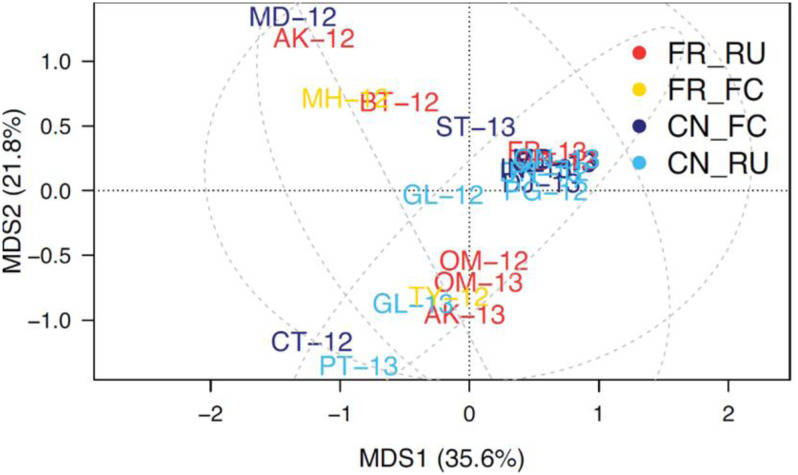
Principal Coordinate Analysis of *flaA* groups across farms. Free-range-re-use (FR_RU), Free-range full cleanout (FR_FC), Conventional Full clean out (CN_FC) and Conventional re-use (CN_RU). (The dotted lines are 95% confidence intervals that group litter practice) (total isolates 757). (Farms are double lettered with year and colour coded for litter practices).

Figure 5 compares the *flaA* groups across farms, (coloured for the litter practices). The principal coordinate analysis, which visualizes the “farm and sample type” separation for *flaA* group profiles, shows a strong separation but does not suggest any “grouping” by practice. Therefore, the analysis of covariance shows that the farm selects for *flaA* distribution; however, (as illustrated in Figure 5) neither farm nor sample type seem to be grouping together, thereby indicating that these are not the primary factors causing the separation. This was supported by a multivariate analysis of similarity (ANOSIM) test (*P* > 0.05). Thus, the four litter practices had no strong influence on *flaA* grouping across the 24 farms.

**Figure 6.**
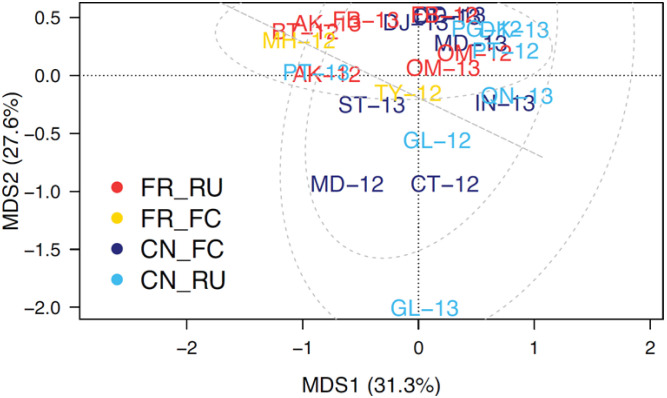
Principal Coordinate Analysis of *Campylobacter* species composition across farms. Free-range-re-use (FR_RU), Free-range full cleanout (FR_FC), Conventional Full clean out (CN_FC) and Conventional re-use (CN_RU). (The dotted lines are 95% confidence intervals that group litter practice), (total isolates 757). (Farms are double lettered with year and color coded for litter practices).

Further analyses considered the *Campylobacter* species balances. Figure 6 separates farms in a multidimensional space depending on the relative frequency of *C. jejuni* and *C. coli*. The farms with higher percentages for *C. coli* (e.g., GL-12 and GL-13) are separated down along the second axis, away from the large cluster of farms near the top of the second axis, and above the dotted line that marks the central coordinate (0,0). Again, the species composition was farm dependent and independent of the farming practice (ANOSIM *P* > 0.05). Additionally, the dominance of *C. jejuni* is apparent in Figure 3, which is based on a PCR analysis of these isolates. Thus, *flaA*-SVR was an effective marker of species segregation for *Campylobacter* sources across all four litter practices. Taken together, this data suggests that something at the individual farms is selecting for *flaA*-SVR group distribution, though it was not “sample type”, “litter practice” or “geographical location”, and that whatever is selecting for *flaA*-SVR can change over time.

### 3.5. Bacteriophages in caeca, litter and soil

**Figure 7.**
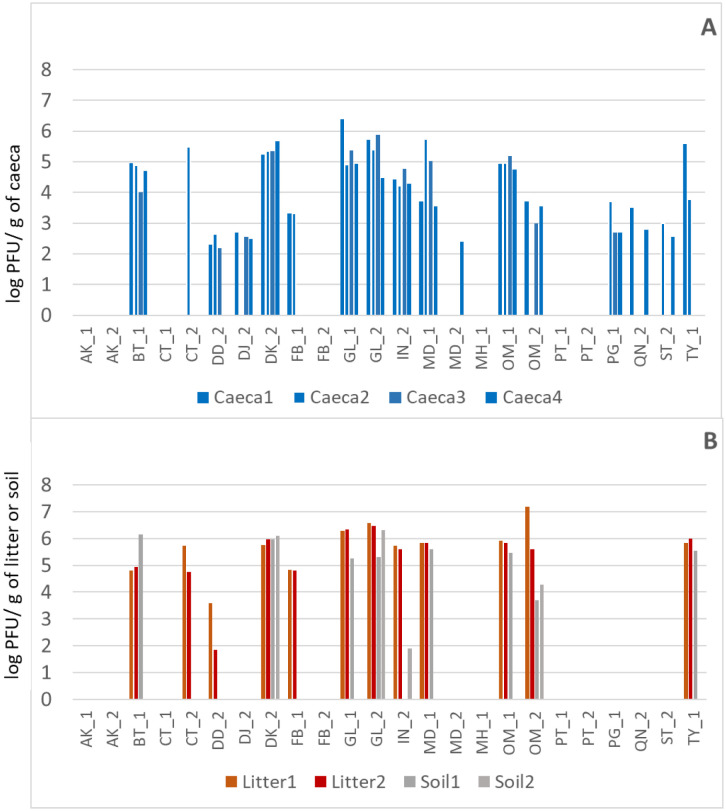
*Campylobacter* bacteriophage levels (with enrichment) in caeca (log PFU/g) across shed across segments C1, C2, C3, C4 (A), litter (log PFU/g) across segments L1, L2 and soil (log PFU/g), across segments S1, S2 (B), for farms Year 1, Year 2.

The levels of bacteriophages in the caeca without enrichment (log 2.3 to log 6.6 PFU/g, Figure S2) and with enrichment (log 2.2 to log 6.4 PFU/g, Figure 7) resulted in enrichment enhancing isolation across numerous farms (rather than increasing bacteriophage levels). As with the caeca *Campylobacter* levels, the levels of bacteriophages isolated across the shed (i.e., segments C1, C2, C3 and C4 (from farms where they were present)) did not markedly vary, though they were not uniform across the shed at times. Bacteriophages were not isolated from litter, soil (Figure 7) and carcasses unless the caeca from the relevant farm was positive, thus suggesting a link between bacteriophage positive birds (i.e., caeca) and the environment (i.e., litter, soil,). Bacteriophages in the litter ranging from log 1.9 PFU/g to log 7.2 PFU/g were isolated from 54% of the farms, in contrast to soil isolation, which was from 38% of the farms (range log 1.9–log 6.1 PFU/g) (Figure 7).

The situation on farm DK_13 was interesting: *Campylobacter* in the caeca was isolated from only one quarter of the shed (shed section C4 at log 7.4 CFU/g); at the same time, high levels of bacteriophages were isolated from the caeca (log 4.23, log 4.04, log 3.48, log 4.08 PFU/g) right across that shed (shed sections, C1, C2, C3, C4 respectively) without enrichment, and slightly higher levels with enrichment (i.e., log 5.22, log 5.34, log 5.34, log 5.68 PFU/g respectively). This demonstrates the natural presence of bacteriophages along with an absence of *Campylobacter* in this shed (except in C4). Another key observation (Figure 7B) was the consistent high bacteriophage levels in the caeca across sequential years irrespective of litter practice on the farms sampled across both years. In GL_12, and GL _13 (conventional litter re-use), the bacteriophage levels ranged from log 6.4 to log 5.9 PFU/g; in OM_12 and OM_13 (free-range litter re-use), they ranged from log 5.2 to log 3.7 PFU/g (free-range litter re-use).

### 3.6. Bacteriophage classification

Transmission electron microscopy of bacteriophage PH388 with a genome size of 145 Kb, is presented in Figure S3. The bacteriophages had genomes sizes from 140–150 Kb, with icosahedral heads and contractile tails characteristic of group 3 phages of the *Myoviridae* family [51]. Now, they are taxonomically classified as *Fletchervirus* in the subfamily *Ecampyvirinae* [74].

## 4. Discussion

A clearer understanding of *Campylobacter*'s persistence, through the broiler production chain, its environmental niche, and its interaction with bacteriophages, can form a multi-stage option to address food-safety [75]. The current study is unique as it adopted a multifaceted approach to assess the *Campylobacter* levels, species diversity, *Campylobacter* populations along with *Campylobacter* bacteriophages in the bird, its immediate environment and the processing plant. For a comprehensive understanding, the former was addressed during commercial farming that assessed the adopted diverse litter practices on the various farms, which ranged from litter re-use to free-range, including conventional. *Campylobacter* survival in the environment is regarded as a paradox, due to its fastidious *in-vitro* growth requirements [76]; however, the successful colonization of the chicken gut by genomic rearrangements is driven by bacteriophage predation [77]. *Campylobacter* is a fragile organism, but its stress response appears to enable the organism to survive diverse conditions, both inside the host and in the environment [78]. Thus, the advances in knowledge on *Campylobacter* colonisation in broilers are also the drivers for the development and implementation of successful on-farm interventions [32].

The current two-year study representing two major integrators in Queensland, Australia provided a broad and practical basis to address the aims of the study: to provide an understanding of *Campylobacter*'s persistence through the broiler production chain, including its environmental niche plus its interaction with bacteriophages. Thus, to ultimately contribute to a multi-stage option, both the on-farm risk management and the potential for bacteriophage biocontrol were targeted. To this end, the bird (on-farm) and the carcass (plant) were simultaneously studied along with the farming environment (litter, soil), thereby adopting a quantitative approach for comparison. On-farm colonisation variability of *Campylobacter* can ultimately impact the levels of contamination across the whole poultry production chain [50].

To the best of our knowledge, few studies have addressed both *Campylobacter* and *Campylobacter* bacteriophages simultaneously under commercial farming, with either one or the other of these factors being the focus of most other studies. We recognised the need for such a study due to our interest in on-farm interventions using *Campylobacter* bacteriophages and developing an informed basis for on-farm risk-management. As discussed later, both native *Campylobacter* and native *Campylobacter* bacteriophage interactions can have a role in caecal *Campylobacter* levels, which influence food-safety. These were the focus of this study and were addressed via a detailed understanding of *Campylobacter* levels, species and populations, simultaneously with bacteriophages. Twenty-four farm samplings represented by 17 commercial farms formed the basis for addressing on-farm *Campylobacter* dynamics. The sequence of farm sampling and the selection of farms was based on the two companies' slaughter schedules at the time and was also representative of the dominant farming practices over the 2-year study period. These practices were full-cleanout, litter re-use and free-range with litter re-use. In contrast, free-range full clean-out practice was represented by two of the 24 farm samplings undertaken and was a limitation of the study. Nevertheless, this practice enriched the study by enhancing the diversity (of isolates and bacteriophages), as they were free-range environments. In the present study, bacteriophages were key to addressing the main focus, namely the on-farm *Campylobacter* dynamics. These diverse Australian farming practices studied formed the basis for addressing on-farm *Campylobacter* dynamics.

Irrespective of the diverse litter practices studied, the mean on-farm *Campylobacter* levels in the caeca were high (log 7.5 to log 8.5 CFU/g) but not statistically different. Additionally, across all litter practices (and farms), *Campylobacter* were evenly distributed across the length of the sheds, as evidenced by their consistent levels across the four categorised shed segments (i.e., C1, C2, C3, C4), thereby representing a full colonised shed across the farms, just prior to final removal for slaughter. Addressing these high *Campylobacter* levels in the bird caeca at this stage contributes to the reduction of *Campylobacter* levels across the process chain and is a key point of risk management.

Across the study, *Campylobacter* was detected during all farm samplings and we have previously reported high *Campylobacter* levels (log 8.0–9.0 CFU/g) in the caeca (independent of farming practice) [9]. In the present study, the quantified *Campylobacter* levels in the caeca were similarly high (log 7.5–8.5 CFU/g) across all sheds. More so, the absence of a negative flock across the current study is not surprising and, if present, would have been detected similar to our previous study. That study reported a negative flock across an entire cycle in three sheds that housed three different litter practices of the 12 cycles studied. That study, which followed six sequential broiler cycles of around 55 days across a year on two separate farms, already demonstrated no impact on the seasonality and timing of sampling on *Campylobacter* detection in a mature flock in sub-tropical Brisbane (Queensland) climates. Additionally, high *Campylobacter* levels have been detected in Polish birds (>log 7.0 CFU/g) [79] and conventionally raised Swedish poultry (log 8.6 CFU/g) [80]. Slightly lower *Campylobacter* levels (log 6.2–6.7 /CFU/g) in the caeca have been reported for both organic and free-range chickens [81]. The *Campylobacter* in the litter microbiome and those in the bird gut can be representative of each other, given the fact that the major component of litter is feces [82]. Thus, rather than the litter (types), as represented by the four farming practices, the chicken (gut), as represented by the flock, appears to be the major influence of *Campylobacter* levels in the caeca. In summary, the two-year study with 17 farms (or a total of 24 independent visits) did not suggest that the litter options or farming practices had an influence on *Campylobacter* levels in the caeca.

Irrespective of litter practices, the mean carcass *Campylobacter* levels at the end of processing ranged from log 2.5 CFU/carcass (free-range full-clean-out) to log 3.2 CFU/carcass (conventional re-use). As with the caeca, carcass *Campylobacter* levels were not influenced by litter options and practices, which included re-use, conventional and free-range. It is possible that rearing systems do not influence *Campylobacter* prevalence on carcasses (and the farm), though an increased genetic diversity (*flaA*-SVR genotypes) is possible with pasture raised flocks, as compared to conventionally raised flocks [83].

The genotypic diversity of *Campylobacter* within broiler sheds can be linked to complex ecological features within the farming environment [84]. The adopted PCR_DGGE contributed to the community profiling of *Campylobacter* isolates sourced across the practices beyond species-level identification. Over the 2-year period, representative *Campylobacter* isolates were categorized based on screening of the *flaA*-SVR to assess the influence of farming practices on the relevant *Campylobacter* populations. PCR screening of the *flaA*-SVR has been adopted for *C. jejuni* [65],[85],[86]. In general, *flaA*-SVR sequences have shed a light on the diverse environmental populations of *Campylobacter* originating from wild geese, starlings and farmed free-range poultry [87].

In the current study, there was a significant association between *flaA*-SVR distribution and the “farm”, which is interesting, as it suggests that something on the farms is selecting for the abundance of certain *flaA*-SVR groups. The SVR region of the *flaA* gene has been shown to be hypervariable and useful in discriminating both *Campylobacter* species and closely related strains [88]. In particular, the *fla*-DGGE method has been successfully used for rapid sub-typing of *Campylobacter* [65]; the *flaA*-SVR region has been proven useful for screening large numbers of *C. jejuni* isolates [89]. Thus, the *fla*-DGGE method was used in the present study to evaluate the strain groupings and ecological analyses of the *Campylobacter* populations. Irrespective of the suggested association, the PCoA, which visualizes the separation of “farm and sample type (caeca, carcass and litter)” based on their *flaA*-SVR group profiles, showed a strong separation, though there was no grouping of the farm litter practices. Therefore, these two aspects are not the factors causing separation. Similarly, there was no significant association between *flaA*-SVR grouping, the three farm regions and litter practices studied. Thus, no factors considered here seemed to influence *flaA*-SVR groupings. In the present study, whatever is selecting for *flaA*-SVR also showed the potential to change over time. It is possible that *Campylobacter* bacteriophages have a role, as all bacteriophages evolve to overcome the host barriers to infection, which is driven by an evolutionary need for co-existence in the chicken gut [48].

*Campylobacter* is known to display genetic instability, maintain diversity and survive in a range of habitats [90], where a change in colonization and species diversity can be driven by *Campylobacter* bacteriophages [43]. An analysis of the multi-locus sequence typing data for *Campylobacter* suggests a genetic exchange between both species, as driven by ecological changes in an agricultural niche with the import of *C. jejuni* alleles by certain clades of *C. coli* [91]. Additionally, recent studies have shown that chicken and ruminant *Campylobacter* strains have contributed to the emergence of an “agriculture associated” *C. coli* lineage, which is important in human disease [92]. Livestock can represent a very different host niche compared to their wild predecessors, thereby enabling recombination across *Campylobacter* species boundaries in situations such as modern intensive poultry farming [93]. There is a need for a broader understanding of both *Campylobacter* species that originate from poultry and their possible role in human disease to target on-farm interventions and risk management.

Over the two years across the four practices, *C. jejuni* (75%) dominated over *C. coli* (25%) based on both PCR and *flaA*-SVR analyses. Moreover, the overall species distribution pattern was not influenced by the regional locations of the farms. Differing environments and geographic distances can influence the stability of *C. jejuni* clones over time [90]. Comparing the caeca, litter and carcasses across the four litter practices, the percentage of *C. jejuni* was not significantly different for the caeca only (its original niche), though it differed between the litter and carcasses. The gut dynamics can influence species diversity in the bird (caeca) [28], which may be the situation seen in the current study.

With respect to carcasses, lower *C. jejuni* percentages (71%) were apparent with both re-use and free-range–re-use, as compared to conventional full-clean-out and free-range–full clean-out (91–95%), in both latter instances, where new bedding was used. Free-range birds [43] and environments [11] are also known to support both *C. jejuni* and *C. coli*. With regards to the litter, a lower percentage of *C. jejuni* was apparent in conventional re-used litter (68%) compared to the rest of the three practices (~100%). *C. jejuni* is known to genetically adapt to survive adverse ecological conditions driven by local environmental pressures [77]. Whilst the reasons for species variabilities observed are not known, a knowledge of species dominance is important to address both the risks and target interventions, especially the key species of concern, namely *C. jejuni*.

Current and potential on-farm interventions to reduce *Campylobacter* numbers have been well reviewed and include anti-*Campylobacter* compounds (bacteriocins), probiotics, vaccines and bacteriophages [94]–[96]. *Campylobacter* bacteriophages can exist along with *Campylobacter* and be a part of the normal microbiota of poultry [51], including in environmentally exposed birds (e.g., free-range) [97]. In the current study, not all farms yielded bacteriophages, with no influence of farming practice, and an overall isolation rate of 54% by direct isolation and 68% with enrichment. This is comparable to the UK, with a 42% isolation rate across the poultry houses [98]. The levels of bacteriophages in the caeca, inclusive of enrichment, ranged from log 2.2 to log 6.6 PFU/g, showing their potential to reach high levels; thus, they are common in broiler farming environments and are a source for biocontrol.

Bacteriophages sourced across this study at the same time as the *Campylobacter* have already shown activity against both *C. jejuni* and *C. coli* across randomly chosen bacteriophage–farm *Campylobacter* host combinations [57]. Bacteriophage diversity in environmentally exposed birds is possible, either due to their external origins or in-gut adaptations to maximise the host advantage [43]. Along with the caeca, bacteriophages were also present in the litter and soil; interestingly, their presence in the litter and soil (and carcasses) was only linked to their simultaneous flock presence (caeca). Thus, the flock status of *Campylobacter* bacteriophages plays a key role in their environmental prevalence due to the host association.

UK studies reported reduced *Campylobacter* levels in the presence of bacteriophages in the caeca (i.e., log 5.1 CFU/g compared to log 6.9 CFU/g in their absence) [99]. However, in the current study, when compared to the rest of the farms, a reduction in the *Campylobacter* levels in the presence of bacteriophages was only detected on a single farm. Farm DK_13 presented an interesting situation: bacteriophages were isolated from the caeca originating across the whole shed from all four categorized shed segments (i.e., C1, C2, C3 and C4 at ~log 4.0–log 5.6 PFU/g in each segment); however, *Campylobacter* was only detected in one segment at the end of the shed (i.e., C4 at log 7.2 CFU/g). This may be a consequence of the gradual and continued elimination of sensitive *Campylobacter* populations along the length of the shed or alternatively the gradual emergence of a bacteriophage resistant population. Such an understanding of native *Campylobacter* bacteriophages is contributory to addressing biocontrol.

In a previous study [55], the same farm (Farm DK_13) demonstrated a late emergence of *Campylobacter* bacteriophages in untreated control chickens, which tested negative a week earlier with reduced *Campylobacter* levels as compared to the bacteriophage cocktail treated birds. Both these separate outcomes that occurred years apart suggested a potential “bacteriophage - farm association”, though no definite proof was possible. The succession of genetically distinct strains of *Campylobacter* through sequential flocks with a bacteriophage influence has been demonstrated in UK poultry [98]. These outcomes demonstrate the complexity of natural on-farm host – bacteriophage interactions and their role in a natural *Campylobacter* reduction in the bird caeca. These outcomes, along with previously undertaken work [55],[57], highlights the need to consider the contribution of native bacteriophages (which also can be farm dependent) during bacteriophage cocktail development. This study provides a basis for understanding natural bacteriophage interactions and contributes to an informed basis for the development (and regulation) of on-farm biocontrol.

Further studies [57] on select bacteriophages from this study and others addressed an in-vitro log reduction (Australian and New Zealand bacteriophages), the evaluation of resistance and safety (i.e., absence of antibiotic resistance and toxin genes etc.) and their performance against select farm campylobacters (including *C. jejuni* and *C. coli*, Australian and New Zealand bacteriophages). These outcomes support future regulation [57] and contribute to the uptake of such interventions [100]. Overall, the outcome of the current study provides an understanding of both *Campylobacter* and *Campylobacter* bacteriophage interactions studied simultaneously on-farm, thereby contributing to the development of informed risk management strategies, including the potential use of bacteriophage biocontrol.

In summary, this multifaceted study adopted a “quantitative approach” to assess both *Campylobacter* and its bacteriophages, simultaneously in the bird (caeca), the immediate farming environment (litter and soil) and the plant (carcasses) across four farming practices during commercial farming. This study, which spanned over two years, demonstrated that the high *Campylobacter* levels in the caeca showed no consistent patterns with *Campylobacter flaA*-SVR population types, species distribution or bacteriophage presence, all of which had no relationship with farming practices. *C. jejuni* was dominant over *C. coli* during the study period.

The *flaA*-SVR group distribution was primarily influenced at an individual farm level and had the potential for change over time; however, that change was not influenced by litter practice. *Campylobacter* bacteriophages naturally occurred in broilers/environments along with *Campylobacter*. More specifically, these natural on-farm host – bacteriophage relationships provide an informed basis for bacteriophage cocktail developments, with a collective understanding of the presence (and role) of native *Campylobacter* bacteriophages.

The study included four key commercial farming practices, thus acknowledging the need to consider these farming choices to arrive at achievable (and practical) on-farm approaches to facilitate the industry uptake and support food-safety. Whilst the key elements of each practice needs to be considered, the outcomes support a common on-farm risk management approach. Thus, the various commercial farming practices should not be a barrier to a unified approach for on-farm *Campylobacter* control. This is of a practical relevance, as some integrator companies can adopt more than one of the studied practices. Overall, the study contributes to addressing overall food-safety, with an understanding of both *Campylobacter* and *Campylobacter* bacteriophage interactions.

This outcome of this study, along with our previous work on an on-farm proof-of-concept study on *Campylobacter* bacteriophages [55] and a detailed study of *Campylobacter* bacteriophages [57] [56], enhance the knowledge base needed for the sustainable adoption of bacteriophage biocontrol by elucidating on-farm *Campylobacter* dynamics. Furthermore, the outcomes of this study on *Campylobacter* dynamics support our previous work on *Campylobacter* and litter practices [9] to provide an informed basis for on-farm risk management and its potential to be supported by *Campylobacter* bacteriophage biocontrol. The study outcomes placed no constraints on farming practice choices, thereby supporting both sustainable (re-use) and emerging (free-range) commercial broiler farming from a perspective of managing on-farm *Campylobacter*.

## 5. Conclusions

In conclusion, this study has shown that *Campylobacter* was isolated across all practices. *Campylobacter* levels in the caeca, which is a key to addressing food safety, were not influenced by the diverse farming practices. Additionally, these farming practices did not influence the *Campylobacter* species types and populations. In contrast, *Campylobacter* bacteriophage isolation was intermittent, with a hint of possible farm association, but not practice dependent. Of interest was the partial non-detection of *Campylobacter* on a single farm though bacteriophages were detected right across that shed. This outcome demonstrates the potential role of native *Campylobacter* bacteriophages in both *Campylobacter* colonisation and levels. Bacteriophages were detected in the environment (litter) only if they were present in the bird (caeca), which is key in demonstrating the close host-phage association and a lack of an environmental role. In summary, there was a lack of farming influence, thereby paving way for a unified approach to *Campylobacter* control across practices. These outcomes contribute to an informed basis for bacteriophage interventions with or without the support of risk management. Future work should focus on generating a better understanding of *Campylobacter* colonisation of the chicken gut, and that includes addressing both *Campylobacter* and *Campylobacter* bacteriophages to progress the control of this key food-safety pathogen in broilers.

## Use of AI tools declaration

No AI tools were used in this study.


