# Supplementary material for: Broiler farming practices using new or re-used bedding, inclusive of free-range, have no impact on Campylobacter levels, species diversity, Campylobacter community profiles and Campylobacter bacteriophages
Source: AIMS Microbiol. 2024 Jan 16;10(1):12–40. doi: 10.3934/microbiol.2024002 (PMC10955168; doi:10.3934/microbiol.2024002)
Supplement: Supplementary file 1 [file microbiol-10-01-002-s001.pdf]

AIMS Microbiology

AIMS Microbiology, 10(1): 12–40.
DOI: 10.3934/microbiol.2024002
Received: 03 August 2023
Revised: 27 November 2023
Accepted: 25 December 2023
Published: 16 January 2024

http://www.aimspress.com/journal/microbiology

Research article

Broiler farming practices using new or re-used bedding, inclusive of free-range, have no impact on *Campylobacter* levels, species diversity, *Campylobacter* community profiles and *Campylobacter* bacteriophages

Helene Nalini Chinivasagam[1,*], Wiyada Estella[1], Damien Finn[1], David G. Mayer[1], Hugh Rodrigues[1] and Ibrahim Diallo[2]

[1] Department of Agriculture and Fisheries, Eco Sciences Precinct, Dutton Park QLD 4102, Australia
[2] Department of Agriculture and Fisheries, Biosecurity Sciences Laboratory, Coopers Plains QLD 4108

* **Correspondence:** Email: nalini.chinivasagam@daf.qld.gov.au; nalinic@me.com; Tel: +61 405606088

Supplementary data

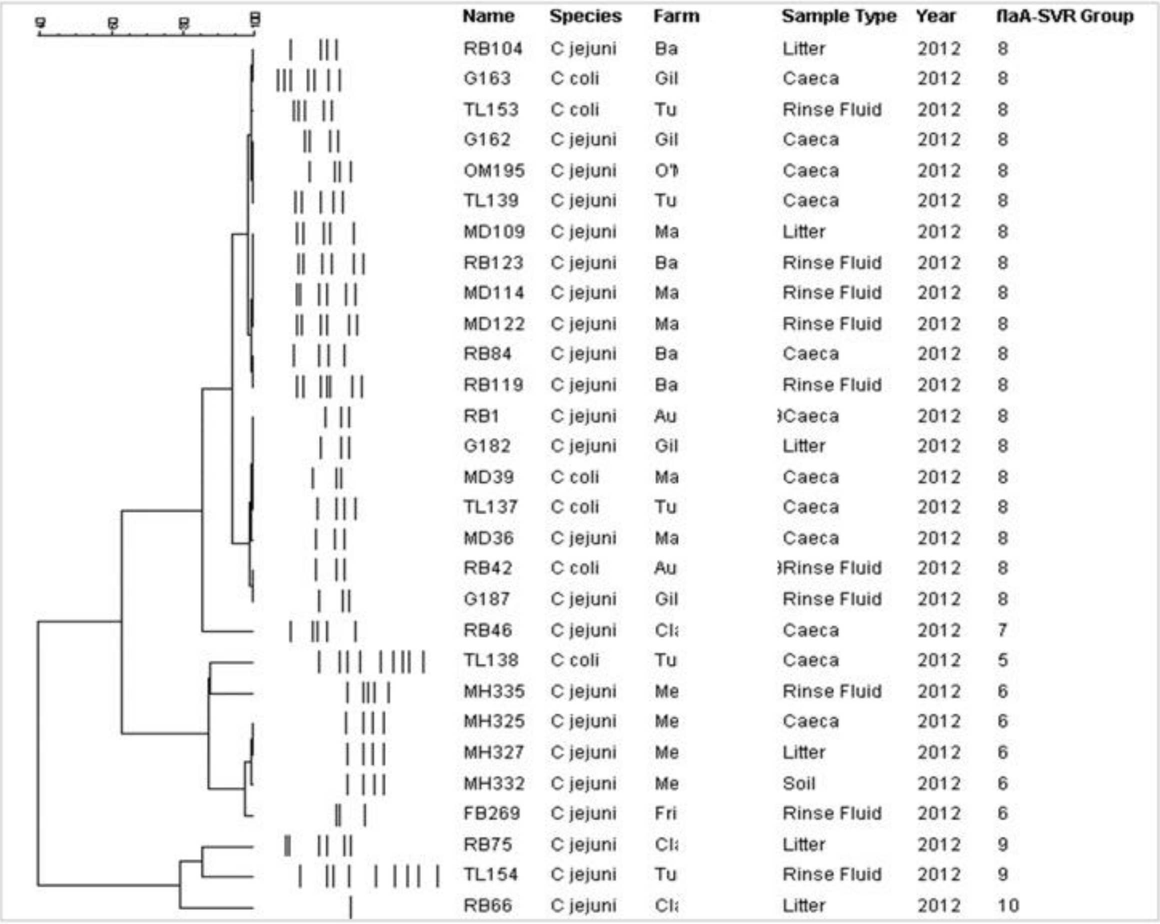

Figure S1. Dendrogram comparing *flaA*-SVR DGGE profiles from year 1 isolates.

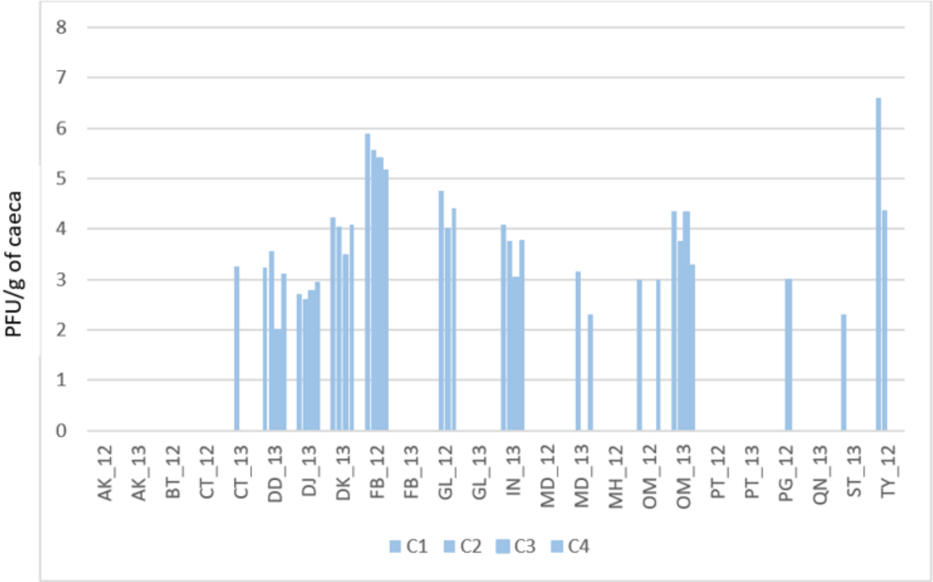

Figure S2. *Campylobacter* phage levels (PFU/g) in caeca (Year 1 and 2) with no enrichment.

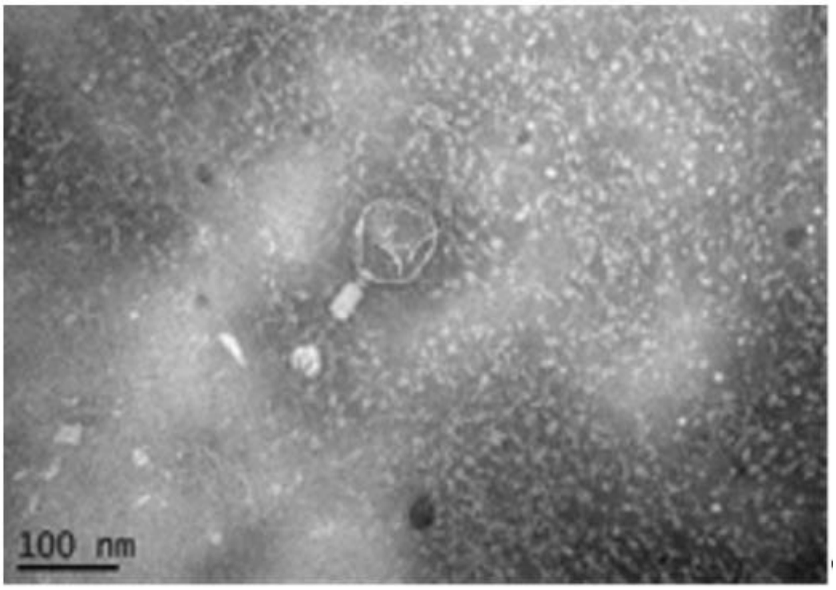

Figure S3. Transmission Electron Microscopy of bacteriophage PH388, genome size 145 Kb (negative staining showing the morphology of the phages).

AIMS Press

© 2024 The State of Queensland (through the Department of Agriculture and Fisheries), licensee AIMS Press. This is an open access article distributed under the terms of the Creative Commons Attribution License (http://creativecommons.org/licenses/by/4.0)
